# SGLT2 inhibitors in diabetic and non-diabetic kidney transplant recipients: current knowledge and expectations

**DOI:** 10.3389/fneph.2024.1332397

**Published:** 2024-04-15

**Authors:** Erietta Polychronopoulou, Fanny Bourdon, Daniel Teta

**Affiliations:** ^1^ Service of Nephrology, Hôpital du Valais, Sion, Switzerland; ^2^ Transplantation Center, Lausanne University Hospital and University of Lausanne, Lausanne, Switzerland

**Keywords:** SGLT2 inhibitor, kidney transplantation, diabetic kidney disease, post transplantation diabetes mellitus, diabetic kidney transplant recipients, CKD - chronic kidney disease

## Abstract

The beneficial effect of sodium-glucose cotransporter-2 inhibitors (SGLT2i) have been shown recently in numerous randomized controlled trials (RCT) and systematic reviews. According to KDIGO guidelines, SGLT2i currently represent a first choice for diabetic patients with chronic kidney disease (CKD). In addition, a recent meta-analysis of 13 large led by the ‘SGLT2 inhibitor Meta-Analysis Cardio-Renal Trialists’ Consortium’ (SMART-C) provided solid evidence of SGLT2i beneficial effects in CKD or in patients with heart failure, with and without diabetes. Collectively, the patients treated with SGLT2i had a decreased risk of CKD progression, acute kidney injury (AKI), end-stage kidney disease (ESKD) or death from heart failure. Whether these cardio-renal benefits should be extrapolated to kidney transplant recipients (KTR) needs to be assessed in further studies. In this article, we report recent data accumulated so far in the literature, looking at the efficacy and safety of SGLT2i in diabetic and non-diabetic KTR. We found encouraging data regarding the use of SGLT2i in KTR with diabetes. These agents appeared to be safe, and they reduced body weight and blood pressure in this group of patients. Potential effects on kidney graft function and survival are yet to be investigated.

## Introduction

SGLT2 inhibitors (SGLT2i), also called gliflozins, inhibit the activity of the Sodium Glucose Cotransporter 2 (SGLT2) in renal tubules, ultimately leading to glucosuria. They were originally thought to only have an effect on glucose control; however large clinical trials have displayed numerous additional advantages, dramatically outperforming initial expectations (1). There are three different gliflozins available in the pharmaceutical market, i.e., empagliflozin, dapagliflozin, and canagliflozin. They all induce glucosuria and thus reduce levels of blood glucose and HbA_1c_ by inhibiting glucose reabsorption in the proximal tubules. SGLT2i also induce sodium excretion which in turn counteracts the tubulo-glomerular feedback (TGF) and to decrease intraglomerular pressure, a crucial mechanism known to foster protective effects on kidney function. As a consequence, SGLT2i reduce albuminuria significantly in diabetic and non-diabetic patients with CKD. This impact on albuminuria is additive to the action of the renin-angiotensin-aldosterone system (RAAS) blockade. With the use of SGLT2i, multiple mechanisms are contributing to the reduction of albuminuria. The most important one is vasoconstriction of the afferent arteriole of the glomeruli, which results in a decrease of the intraglomerular pressure and hyperfiltration (1). Additional effects of the SGLT2i include reduction of inflammatory marker levels of IL-6, TNF-alpha, IFNγ, NF-κβ, TLR-4, and TGF-β and improvement of mitochondrial function (2). These mechanisms may contribute to limit inflammation, fibrosis, and oxidative stress in heart and kidneys tissues. It is important to note that all these modifications seem to result from consequences of metabolic and hemodynamic effects of SGLT2 co-transporter inhibition (2–5).

## Hyperfiltration pathophysiology in renal transplantation

Kidney transplantation is characterized by glomerular hyperfiltration, which is also observed in various clinical settings associated with nephron reduction. Hyperfiltration results from afferent arteriolar vasodilation, and/or by efferent arteriolar vasoconstriction secondary to the activation of the RAAS, consequently leading to glomerular hypertension and thus glomerular injury in the remaining nephrons (4, 6). In KTR, hyperfiltration is clearly an adverse factor leading to unfavorable long-term kidney outcomes in this group of patients already subjected to injuries through immunological and non-immunological mechanisms (7). Since SGLT2i are efficiently reducing glomerular hyperfiltration, it is anticipated that these agents may prove instrumental to improve kidney allograft outcomes.

## SGLT2i and CKD recommendations

Recent RCT in native CKD have shown a large range of clinical benefits of SGLT2i, especially cardio renal protective effects in patients with and without type 2 diabetes mellitus (8). In addition, the EMPEROR-Reduced randomized placebo-controlled trial, which aimed to explore the impact of empagliflozin in patients with reduced ejection fraction across a broad range of different kidney functions, has demonstrated a significant decrease in cardiovascular deaths and heart failure hospitalizations, in favor of the empagliflozin versus the placebo group (9). According to the above evidence, SGLT2i now constitute a first-line treatment for diabetic patients with CKD, together with metformin, RAAS blockade and statins, as recommended in the KDIGO guidelines 2022 (10). For all patients, lifestyle adaptation and control of risk factors are the basis of this approach, including diet, physical activity, smoking cessation, and body weight control. In addition to these basic measures, there is a range of proven drug treatments, depending on the patient’s comorbidities, including SGLT2i. For patients with diabetes and CKD, a combination of metformin, if estimated glomerular filtration rate (e GFR) > 30ml/min/1.73m^2^ and SGLT2i (introduced if eGFR > 20ml/min/1.73m^2^ and continued until dialysis or transplantation), is recommended. Statin therapy is also recommended. Depending on other comorbidities, the addition of RAAS inhibitors is a first-line treatment for patients with hypertension or albuminuria. If glycemic control is unsatisfactory despite SGLT2i and metformin, or if their use is contraindicated, glucagon-like peptide-1 (GLP-1) receptor agonists may be used. Non-steroidal mineralocorticoid receptor antagonists (ns-MRA) can be added to first-line treatments in diabetic patients with a high risk of CKD progression, such as persistent albuminuria > 3g/mol.

What is more astonishing is that SGLT2i have been proven to retard the decline of eGFR in non-diabetic individuals with proteinuria (6). However, according to initial studies in CKD, there were safety concerns regarding severe urinary tract infections (UTI) and vaginal infections in women. This is why the extension of these clinicals benefits to the KTR population needs to be assessed in further studies.

## Efficacy and safety of SGLT2i in diabetic kidney transplant recipients

The use of SGLT2i in KTR could be of great advantage, especially because the characteristics of this group of patients, with significant cardiovascular risk, and chronic kidney allograft disease. These patients especially suffer from prevailing long-term kidney allograft lesions usually associated with hyperfiltration, such as arteriolar hyalinosis and focal segmental glomerulosclerosis (1, 11). Halden et al. (12), carried out a first prospective placebo controlled RCT evaluating the efficacy and the safety of empagliflozin 10 mg, once a day, in KTR with post-transplant diabetes mellitus (PTDM). There were no significant differences between empagliflozin and placebo regarding adverse events, immunosuppressive drug levels, and eGFR decline. The group of empagliflozin-treated patients showed a statistically significant reduction in HbA1C and body weight, compared with the placebo group (-0.2% vs. +0.1%, p=0.025 and -2.5 kg vs. +1.0 kg, respectively, p=0.014). There was no significant difference between the two groups, concerning incident UTI (12). This study was however limited by the small number of participants (n=44) and the short follow-up of 24 weeks. In addition, several observational studies (**Table 1**) also looked at SGLT2i in KTR with type 2 diabetes (T2DM) or PTDM (24). These studies demonstrated that patients with SGLT2i presented a lower glucose level and lower body weight and blood pressure. No difference in adverse events was noted when KTR were compared with native CKD patients.

**Table 1:** Observational studies so far and ongoing (1, 13).

Study	Population	Intervention	Results with SGLT2i
Fructuoso et al., 2023 (14).	Observational, n= 339 KTR with T2DM or PTDM		Significant reductions in body weight, BP, fasting glycaemia, HbA1c, UPCR. The most frequent adverse event was UTI in 14%.
Rajasekeran et al., 2017 (15)	Case series (4 SPKT, 6 KTR) KTR or SPKT on canagliflozin	Canagliflozin	Reduction of HbA1c −0.84% Reduction of body weight −2.14 kg Reduction of BP −6.4mmHg No UTI or mycotic infection
AlKindi et al., 2020 (16).	Case series (8 KTR) KTR on SGLT2i (T2DM or PTDM)	Empagliflozin (6 patients), dapagliflozine (2 patients)	-6 months follow-up: Decrease in body weight -4kg -12 months follow-up: Decrease of HbA1c Stable eGFR Stable BP One episode of UTI
Attallah and Yassine 2019 (17)	Case series (8 KTR) KTR on empagliflozin (T2DM or PTDM)	Empagliflozin	-12 months follow-up: Reduction of HbA1c Slight reduction in eGFR, then stabilized Reduction of proteinuria (UPCR) of 0.6 g/day. Reduction of body weight - 2.4 kg. Two cases of UTI
Schwaiger et al., 2019 (18)	Prospective interventional study (14 KTR) KTR with insulin	Empagliflozin	-4 weeks follow-up: Reduction of eGFR by 7.5 ml/min/1.73 m2. Three cases of UTI and one case of uncomplicated balanitis. -12 months follow-up: Stable HbA1c. Reduction of body weight -1.6 kg
Mahling et al., 2019 (19)	Prospective case series (10 KTR) Stable KTR	Empagliflozin	-12 months follow-up: No change in eGFR Reduction of body weight −1.9 kg Low rate of UTI and other side effects
Shah et al., 2019 (20)	Prospective descriptive study (24 KTR) Stable KTR with T2DM or PTDM	Canagliflozin	-6 months follow-up: Decrease of body weight -2.5kg Decrease of: SBP -8mmHg, DBP -2mmHg HbA1c reduction -0.9% No UTI increase
Song et al., 2020 (21)	Observational retrospective (50 KTR) KTR, eGFR >30 ml/min/1.73 m^2^ with T2DM or PTDM	Empagliflozin (*n* = 43), canagliflozin (*n* = 6) or dapagliflozin (*n* = 1)	-6 months follow-up: Decrease of body weight −2.95 kg No increase of UTI Stable renal function
Lim et al., 2022 (22)	Observational retrospective (226 KTR) KTR with T2DM on SGLT2i		Lower risk for all-cause mortality, death-censored graft failure and serum creatinine doubling eGFR stable
Lemke et al., 2022 (23)	Observational retrospective (39 KTR) KTR on SGLT2i (T2DM or PTDM)	Canagliflozin (*n* = 12), dapagliflozin (*n* = 3), empagliflozin (*n* = 24)	-12 months follow-up: Stable renal function UTI was the most common adverse event HbA1c reduction -1%

AKI, acute kidney injury; BP, Blood Pressure; DBP, diastolic blood pressure; eGFR, estimated Glomerular Filtration Rate; RCT, Randomized Control Trial; KTR, kidney transplant recipient; SBP, systolic blood pressure; PTDM, Post Transplant Diabetes Mellitus; SPKT, Simultaneous pancreas-kidney transplantation; UPCR, Urine Protein Creatinine Ratio; UACR, Urine Albumin creatinine Ratio; UTI, Urinary Tract infection.

There are actually six ongoing RCT (**Table 2**) looking at the use of SGLT2i in KTR. First, the CREST-KT (25), which is a single center double-blind RCT in 72 patients, with a follow-up of 18 months. A second study from the University of Toronto is a randomized double-blind study, dapagliflozin versus placebo, looking at blood pressure as a primary outcome in patients with preexisting diabetes (26). A third large randomized, placebo-controlled trial study including 330 patients during 3 years of follow-up from Oslo University Hospital is investigating the effect of SGLT2i in KTR looking at preservation of eGFR, reduction of interstitial fibrosis in the kidney transplant, and metabolic risk factors for graft failure such as visceral obesity, glucose intolerance and blood pressure (27). Another ongoing study also from the University of Toronto plans to determine the short-term efficacy, mechanisms, and safety of a combination between dapagliflozin and semaglutide (GLP-1 receptor agonist) over 12 weeks in 20 KTR, with and without T2DM, i.e. the HALLMARK study (28). Another, randomized single-blinded controlled trial from the University of Sao Paulo General Hospital, aims to evaluate the effect of dapagliflozin on the renal functional deterioration of KTR with or without diabetes (29). The authors intend to enroll 220 KTR, in order to evaluate mean eGFR differences, between baseline and one year after randomization. Studies are also anticipated in non-diabetic KTR. Finally, the RENAL LIFECYCLE Trial, a multicenter RCT from the University Medical Center Groningen, which has as an objective to establish the reno- and cardioprotective efficacy and safety of dapagliflozin in patients with severe CKD, including KTR patients (30).

**Table 2:** Randomised clinical trials so far and ongoing.

Study	Population	Intervention	Results with SGLT2i
Halden et al., 2019. (12)	*n* = 44 (22 patients on empagliflozin) PTDM with KTR >1 year, eGFR >30 ml/min/1.73 m^2^	Empagliflozin versus placebo	• Reduction of HbA1c: −0.2% • Reduction of body weight • No changes in BP or eGFR • No interactions observed with immunosuppressive drugs • One case of urosepsis and one mycotic infection
CREST-KT ongoing (25)	72 KTR with (n=36) and without (n=36) T2DM	Empagliflozin versus placebo	Primary outcomes: •Change in kidney function as measured by albuminuria •Change in cardiac structure •Change in blood insulin level •Change in fasting blood sugar •Number of UTI and genital infections
INFINITI 2019 ongoing (26)	52 KTR with T2DM or PTDM	Dapagliflozin versus Placebo	Primary outcome: determine if dapagliflozin is superior to placebo in reduction of BP in KTR.
Can Dapagliflozin Preserve Structure and Function in KT? Ongoing (27)	330 KTR	Dapagliflozin versus Placebo	Primary outcome: •Effect of Dapagliflozin on the GFR slope in KTR. • Difference in eGFR slope between groups
The Efficacy, Mechanism & Safety of Sodium Glucose Co-Transporter-2 Inhibitor & GLP-1 Receptor Agonist Combination Therapy in Kidney Transplant Recipients (HALLMARK) Ongoing (28)	20 KTR, with and without T2DM	Dapagliflozin Semaglutide	Primary outcome: • Mechanisms and safety of 12 weeks of dapagliflozin and semaglutide combination therapy.
Effect of adding dapagliflozin to allograft dysfunction of renal transplanted patients Ongoing (29)	220 KTR, with and without T2DM	Dapagliflozin versus Placebo	Primary outcome: •Estimate the mean delta difference of the eGFR, between baseline and one year after randomization.
The RENAL LIFECYCLE Trial: A RCT to Assess the Effect of Dapagliflozin on Renal and Cardiovascular Outcomes in Patients with Severe CKD (30)	1500 patients -with advanced CKD, i.e. an eGFR ≤25mL/min*1.73m2 -on dialysis with a residual diuresis >500 mL/24h (at least 3 months after start of dialysis) -KTR and an eGFR ≤45mL/min/1.73m2 (at least 6 months after transplantation)	Dapagliflozin 10 mg/day or matching placebo	Combined endpoint of all-cause mortality, kidney failure, and hospitalization for heart failure in the overall study population

## Risks and concerns

### Urinary tract infections

The main concern with SGLT2i in the kidney transplant community is the potential increased risk for UTI. Data accumulated so far, do not show a significant difference in the occurrence of UTI in patients using SGLT2i versus those with no SGLT2i. In most studies, patients selected for the use of SGLT2i were started after more than 1-year post-transplantation, due to clinician’s main concern regarding a possible decline in eGFR and the incidence of UTI during the first year after kidney transplantation (24).

### Acute kidney injury

SGLT2i might result in AKI by multiple mechanisms, such as depletion of effective volume because of excessive diuresis, or because of significant decrease in trans-glomerular pressure, especially in patients on RAAS blockade. Another possible mechanism is the hypoxic injury, associated to elevated distal tubular transport, particularly with the simultaneous use of agents already known to impair oxygen transport to the kidney medulla, such as non-steroidal anti-inflammatory drugs or radiographic iodine contrast agents. Thus, in KTR, clinicians must be careful on the importance of maintaining blood volume; in particular, patients must be informed that, in case of volume-depletion situations, for instance vomiting, diarrhea, SGLT2i should be discontinued (31).

### Increased risk for amputations

The Canagliflozin Cardiovascular Assessment Study (CANVAS) and the Canagliflozin Cardiovascular Assessment Study-Renal (CANVAS-R) demonstrated that canagliflozin increased leg and foot amputations in research participants compared with placebo (6.3 vs 3.4 per 1,000 patient-years) (32). Data from studies in KTR until now, have not shown an increase in the incidence of amputations, although studies were underpowered to look at this outcome (12).

### Ketoacidosis

SGLT2i, may cause euglycemic ketoacidosis; however, this risk is extremely low.

It is not advised to use SGLT2i in patients who have predisposing factors such acute gastroenteritis or insulin pump failure (33). No episode of ketoacidosis was reported in the study conducted by Halden et al. (12). For type1 diabetes (T1D) patients, SGLT2i potentially address some of the unmet needs associated with T1D. They improve glycaemic control and induce weight loss,increasing hypoglycemia. However, because of side effects, the european recommendation for the use of SGLT2i on T1D was withdrawn. Further studies are needed to determineSGLT2i safety in T1D and to define the type of patient who can benefit most from these medications (34).

## Discussion

Most of the existing evidence concerning the application of SGLT2i in kidney transplantation is accumulated mostly from observational studies and only one small RCT. Ongoing RCT in this population are in progress. Although the evidence remains insufficient, these data, mostly observational, encourage physicians to use SGLT2i in diabetic KTR. The evidence shows encouraging data regarding the incidence of UTI and the decrease of body weight and blood pressure (1). Long-term outcomes regarding renal graft function and survival are still awaited. The data show that the use of SGLT2i in diabetic KTR results in lower levels of HbA1c without an increased risk for UTI or euglycemic ketoacidosis. The absence of significant drug-drug interaction with the immunosuppressive treatment and the advantageous impact of SGLT2i including weight loss, make SGLT2i an appealing treatment option for KTR (24). Two recent systematic review and meta-analysis also indicate that although studies with extensive follow-up are required in KTR with or without PTDM receiving SGLT2i so as to evaluate their potential benefits in prevention of allograft dysfunction, it is still reassuring that no clinically significant acute falls in the eGFR were detected (35, 36).

The studies in KTR, appear to show similar results than in the native CKD population. SGLT2i in KTR may cause an acute temporary decline of eGFR, which is thought to be associated with SGLT2i induced afferent arteriolar vasoconstriction. There is evidence that even in the denervated kidney allograft, SGLT2i have the same impact in natriuresis that eventually results to increased tubulo-glomerular feedback and afferent arteriolar vasoconstriction (24). Further research is required to explore the potential impact of reducing intraglomerular hypertension and hyperfiltration on proteinuria/albuminuria and on preservation of eGFR in KTR. The studies so far, offer valuable insights which may stimulate to the development of additional studies focused on establishing the safety and effectiveness of these medications, along with their potential benefits in terms of cardiac and renal protection for KTR, in diabetic and non-diabetic patients. Although the evidence remains insufficient, these data, mostly observational, should encourage renal transplant physicians to use SGLT2i in diabetic KTR, after the first 12 months of follow-up since kidney transplant surgery, mainly for the effect on blood pressure and weight. We recommend using SGLT2i in this setting. Patients should be instructed to maintain sufficient, if not overhydration, in order to prevent eGFR to suffer from hemodynamic effects of SGLT2i. In addition, patients should be counseled regarding perineal hygiene measures to prevent UTI. We do not recommend using SGLT2i in recipients who underwent repeated UTI. In non-diabetic KTR, data are yet insufficient to recommend the use of these drugs, although their great potential may see the light in the near future. Novel and ongoing RCT in this population are expected to provide positive hard clinical outcomes, especially renal allograft survival in the near future.

## Author contributions

EP: Writing – original draft, Writing – review & editing. FB: Writing – review & editing. DT: Supervision, Writing – original draft, Writing – review & editing.
